# A Descriptive Account of a Rare Case of Mal-Formation in a Child

**Published:** 1821-03

**Authors:** Joseph Howell

**Affiliations:** Member of the Royal College of Surgeons in London.


### *A Descriptive Account of a rare Case of Mal-formation in a Child.* By Joseph Howell, Member of the Royal College of Surgeons in London.

AT the termination of the eighth month of her pregnancy, a young woman was delivered of her first child, the subject of the following singular case of *lusus naturæ.*

Through an opening, formed by a deficiency of the upper part of the parietes of the abdomen, six inches in circumference, protruded the liver, gall-bladder, spleen, prancreas, the stomach, and the whole of the small intestines. The edges of the opening through which these viscera protruded, was smooth and well-formed, being very similar in appearance to the prepuce embracing the glans penis. The upper and greater part of this mass of viscera was surrounded by a muscular membrane, separable into three distinct thin layers, but destitute of common integuments, coming from the edge of the opening at its upper part. This covering hung loose, not adhering to the parts over which it was reflected, and did not extend low enough to cover the protruded intestines, the coats of which were protected by little detached slips of muscle, covering those convolutions only which were external. The *two* umbilical arteries arose from the left common iliac, and, emerging from the cavity of the abdomen, entered the membrane hanging over the viscera at its left edge; proceeding between the layers of muscle, a little upwards, they came in contact with the umbilical rim which descended from the anterior part of the broad ligament of the liver; passing out from the said covering, the vessels formed the chord of the usual appearance and length. The thorax and head were examined, and found perfect; and the child, which was a male, was well-proportioned, and of the common size. It lived twenty-four hours, and was fed once; but passed nothing from its bowels. The handling of the protruded viscera did not seem to give pain, yet at other times the child cried lustily. It died quietly, without seeming to experience any suffering.

*Wimbledon; Jan.* 19, 1821.

